# Structural changes of bacterial nanocellulose pellicles induced by genetic modification of *Komagataeibacter hansenii* ATCC 23769

**DOI:** 10.1007/s00253-019-09846-4

**Published:** 2019-04-29

**Authors:** Paulina Jacek, Małgorzata Ryngajłło, Stanisław Bielecki

**Affiliations:** Institute of Technical Biochemistry, Lodz University of Technology, B. Stefanowskiego 4/10, 90-924 Lodz, Poland

**Keywords:** *Komagataeibacter*, Motility, Cell division, Bacterial nanocellulose

## Abstract

Bacterial nanocellulose (BNC) synthesized by *Komagataeibacter hansenii* is a polymer that recently gained an attention of tissue engineers, since its features make it a suitable material for scaffolds production. Nevertheless, it is still necessary to modify BNC to improve its properties in order to make it more suitable for biomedical use. One approach to address this issue is to genetically engineer *K. hansenii* cells towards synthesis of BNC with modified features. One of possible ways to achieve that is to influence the bacterial movement or cell morphology. In this paper, we described for the first time, *K. hansenii* ATCC 23769 *motA+* and *motB+* overexpression mutants, which displayed elongated cell phenotype, increased motility, and productivity. Moreover, the mutant cells produced thicker ribbons of cellulose arranged in looser network when compared to the wild-type strain. In this paper, we present a novel development in obtaining BNC membranes with improved properties using genetic engineering tools.

**Electronic supplementary material:**

The online version of this article (10.1007/s00253-019-09846-4) contains supplementary material, which is available to authorized users.

## Introduction

Bacterial nanocellulose (BNC) synthesized by bacteria from *Komagataeibacter* genus is one of the most intensively studied biopolymers due to its unique properties such as high mechanical strength, chemical stability, hydrophilicity, crystallinity, and biocompatibility. BNC is the most abundant renewable organic material produced in the biosphere (Gallegos et al. 2016; Ryngajłło et al. 2018). Because of these properties, BNC has a wide range of potential biomedical applications, including wound dressings, medical implants, drug delivery, vascular grafts, and scaffolds for tissue engineering (Svensson et al. 2005; de Oliveira Barud et al. 2016; Picheth et al. 2017). In recent years, many studies have been carried to develop BNC scaffolds for biomedical use (Wu et al. 2016; Cielecka et al. 2018; Jacek et al. 2018). Nevertheless, the main problem for tissue engineering is the limited porosity of the BNC that makes the ingrowth of the eukaryotic cells is impossible.

The properties of a BNC membrane closely depend on its morphology. One of the recent approaches aiming to change the morphology of BNC involves the regulation of bacterial cell movement, by using different types of electromagnetic fields (Sano et al. 2010; Baah-Dwomoh et al. 2015; Fijałkowski et al. 2015). Use of electrokinetic force to control bacterial motion of *Komagataeibacter xylinus* allowed for the differentiation of orientation and organization of bacterial nanocellulose fibers (Baah-Dwomoh et al. 2015). Furthermore, the intrinsic cell motility was shown to be important for pellicle formation in many species (Hölscher et al. 2015). Modification of bacterial nanocellulose structure has mainly been achieved by chemical or mechanical modifications of the cellulose matrix or via changing culturing conditions, while there are no successful results in changing the BNC architecture using genetic engineering tools (Chanliaud and Gidley 1999; Luo et al. 2008; Lee et al. 2014). Nevertheless, genetic engineering remains a promising approach to enable a fine control over nanocellulose synthesis towards production of diverse BNC-based biomaterials.

The studies, which involved genetic modifications of the *Komagataeibacter* strains, focused mainly on the genes affecting efficiency of cellulose biosynthesis (Jacek et al. 2019). These genetic modifications included the expression of a foreign gene, the gene disruption, or gene overexpression. One of the most impressive examples of these genetic modifications is the *K. xylinus* mutant which uses lactose from whey as a carbon source, producing 28-fold more cellulose from lactose than the wild-type strain (Battad-Bernardo et al. 2004). Another successful application of recombinant strains for bacterial nanocellulose production from wastes is a mutant of *K. xylinus* with enhanced ability to produce BNC from a hydrolysate of a potato pulp (Zhong et al. 2013).

Bacterial motility is a complex phenomenon playing diverse roles in biofilm formation. Most of the motile bacteria move with flagella, which is an essential multifunctional organellum involved in the initial stages of biofilm formation (Bogino et al. 2013). Bacterial cells can move through the liquid environment or translocate over the surface of solids, using various mechanisms. Bacterial chemotaxis is a kind of movement by which microorganisms efficiently and rapidly react to the changes in the chemical composition of their surroundings, approach chemically beneficial environments, and avoiding unfavorable ones (Tamar et al. 2016). The core components of the chemotaxis mechanism are universal for all motility systems and highly conserved among bacteria; however, some, often complex, variations have been identified (Szurmant and Ordal 2004). According to literature data, strains of the *Komagataeibacter* genus exhibit chemotactic movement (Basu et al. 2018).

In Gram-negative bacteria, cellular motility and cell division are described as one of the essential factors for biofilm formation (Pratt and Kolter 1998; Merritt et al. 2007; Niba et al. 2007). Many reports suggest that proteins MotA/MotB, ExbB/ExbD, and TolQ/TolR which form transmembrane proton channels are important in biofilm formation (Abbas et al. 2007; Søgaard-Andersen 2011; Guttenplan and Kearns 2013; Islam and Mignot 2015; Santos et al. 2015). MotA and MotB are membrane-bound proteins, which form a heterohexamer MotA_4_/MotB_2_ and are indispensable for bacterial motility. MotA/MotB works as the stator of the bacterial flagellar motor to couple proton influx with torque generation (Morimoto et al. 2010). It has been reported that Gram-negative bacterium *Myxococcus xanthus* that glides over surfaces without the aid of flagella has AglS, AglR, and AglQ, which are MotA/MotB homologs (Søgaard-Andersen 2011). Flagellar motor proteins MotA/MotB and gliding proteins AglQ-AglL/AglS share homologies with the components of the Ton and Tol systems (ExbB/ExbD and TolQ/TolR)—systems that also form proton channels and utilize a proton-motive force (PMF). However, these proteins are not involved in motility but in the active transport of substrates across the outer membrane (Cascales et al. 2001; Sun et al. 2011; Gray et al. 2015). ExbB and ExbD proteins form a complex, which is a component of the energy transducing Ton system, and which functions to harness the energy of the PMF at the cytoplasmic membrane to support active transport of iron-siderophore complexes and vitamin B12 across the outer membrane in Gram-negative bacteria (Andrews et al. 2003; Postle and Kadner 2003; Noinaj et al. 2010). TolQ and TolR together with TolA form a complex in the cytoplasmic membrane that plays an essential role in maintaining outer membrane integrity and cell division (Yeh et al. 2010; Teleha et al. 2013).

Currently, there is no information explaining potential role of MotA/ExbB/TolQ and MotB/ExbD/TolR in *Komagataeibacter* genus. Moreover, little is known about the role of motility and cell division on bacterial nanocellulose biosynthesis and properties. In 1976, Brown et al. first characterized the movement of *Komagataeibacter hansenii* cells in relation to the BNC biosynthesis (Brown et al. 1976). They observed that cell motility rate correlates with the actual biosynthesis and extrusion of bacterial nanocellulose microfibrils. Consequently, they hypothesized that cell movement is caused by the propulsion force of BNC microfibrils secretion by the bacterium (Brown et al. 1976).

Herein, for the first time, *K. hansenii* ATCC 23769 was modified by using genetic engineering tools, in order to obtain the loosened structure of BNC membranes. In this study, we created and investigated two overexpression mutants; *motA+* and *motB+*. Moreover, to get more information about the functions of the MotA and MotB proteins, we investigated their subcellular location. Our results expanded the understanding the role of *K. hansenii* motility and cell morphology in BNC structure architecture.

## Materials and methods

### Bacterial cell culture conditions and growth media

*Komagataeibacter hansenii* ATCC 23769 was cultivated in SH medium (Schramm and Hestrin medium) at 30 °C. The SH medium for *K. hansenii* cultivation was used in two forms: liquid—for agitated and stationary cultures and with 2% (*w*/*v*) agar (Difco, USA)—for plate cultures. One-liter culture medium contained 20.0 g glucose (POCH, Poland), 5.0 g yeast extract (BTL, Poland), 5.0 g bacterial peptone (BTL, Poland), 2.7 g sodium phosphate dibasic (Chempur, Poland), 1.15 g citric acid (Chempur, Poland), and 0.5 g magnesium sulfate (Chempur, Poland). The pH was adjusted to 5.7 with 80% acetic acid (Chempur, Poland) before sterilization.

### Plasmid construction and transformation

For *motA+* and *motB+* mutants, pTI99 plasmid (Hokkaido University) was used (Table 2). Initially, *motA* and *motB* were amplified from the genomic DNA of *K. hansenii* ATCC 23769 with primers pTI-motA_for, pTI-motA_rev primers and pTI-motB_for, pTI-motB_rev primers, respectively (Table S1). The amplified DNA fragments and pTI99 plasmid were treated *Bam*HI and *Hind*III and ligated. The resulting constructs were transformed using the heat shock method into *E. coli* TOP10F’ cells (Froger and Hall 2008). Then, *K. hansenii* ATCC 23769 was transformed with control pTI99, pTI99-motA, pTI99-motB by electroporation, in accordance with the method used in previous studies (Jacek et al. 2018). The cells were incubated at 30 °C for 3 h, diluted, and transferred to SH/agar plates containing ampicillin (200 μg/mL) for screening of recombinants (Fig. S4).

### MotA-GFP and MotB-GFP fusion protein generation and expression

To construct plasmids carrying the *motA-gfp* and *motB-gfp* fusion genes, the *motA* and *motB* genes were amplified by PCR using the *K. hansenii* ATCC 23769 genomic DNA as a template and the motA_gfp_F, motA_gfp_R primers and motB_gfp_F, motB_gfp_R primers, respectively. The amplified DNA fragments were treated with *Eco*RI and *Bam*HI (for *motA*), *Kpn*I, and *Bam*HI (for *motB*), and then ligated with a pTIE plasmid digested with the same restriction enzymes. All plasmids, including the control vector pTIE-GFP, were introduced into *K. hansenii* ATCC 23769 by electroporation (Hall et al. 1992).

### Bacterial growth curves

To monitor cell growth and survival, viable cell counts were determined by serial dilution. A single colony was inoculated into a test tube containing 5 mL of liquid SH medium with/without ampicillin and grown at 30 °C in static conditions. Every 24 h, 1.5% of cellulase (Sigma-Aldrich, Denmark) was added and incubated for 3 h and periodically stirred. Then, serial dilutions were made in 0.9% NaCl and spread on SH with agar plates with/without ampicillin. The experiments were carried out in triplicate.

### *K. hansenii* ATCC 23769 cell length determination

Liquid cultures in SH medium with/without 200 μg/mL ampicillin of four tested *K. hansenii* ATCC 23769 strain variants (wild-type, control, *motA+*, and *motB+* mutants) were used for cell length determination. During 6 days of incubation, 10 μL portions of cells suspension were fixed to the microscope slide and stained with crystal violet, every 24 h. From each slide, 20 pictures were taken, with use of light microscope Olympus BX 51 (Olympus, Japan) under 400 times magnification. Cells lengths were measured with *Makroaufmassprogram* software of at least 10 cells randomly chosen from each picture. Distribution of obtained values comprises at least 300 measurements of individual cells for each strain.

### *E. coli* TOP10F’ cell length determination

Liquid cultures in LB medium supplemented with/without 100 μg/mL ampicillin of four tested *E. coli* TOP10F’ strain variants (wild-type, control, *motA+*, and *motB+* mutants) were used for cell morphology determination. After 12 h of incubation, 10 μL portions of cells suspension were fixed to the microscope slide and stained with crystal violet and observed in light microscope Olympus BX 51 (Olympus, Japan) under 1000 times magnification. Representative pictures were taken for each strain.

### Swarming motility assay

Pre-cultures of three tested *K. hansenii* ATCC 23769 strain variants (wild-type, control and mutant) were diluted to reach optical density of 0.1 at 600 nm. Next, 2 μL portions from each equilibrated culture were inoculated onto five 0.3% agar SH plates containing 2% (*v*/v) cellulase (from *Trichoderma reesei* ATCC 26921, Sigma-Aldrich, Germany). Spots of mutant culture were accompanied by spots of wild-type and control strains on the same plates. After 3 days of incubation the diameters of colonies were measured. Measurement was repeated at 24 h interval two more times. Experiments were performed in five replicates and the results are given as means.

### Bacterial nanocellulose biosynthesis

Liquid culture for inoculum was prepared by transferring a single bacterial colony grown on SH agar seed medium into 5 mL of a liquid SH medium, both with or without antibiotics, which was then incubated at 30 °C for 4 days. Five percent of the inoculum culture was introduced into a 250-mL Erlenmeyer flask containing 50 mL of SH medium. The culture was then incubated statically at 30 °C for 7 days. After incubation, membranes were picked and soaked in 2% NaOH solution for one night, and, next in 1.5% acetic acid solution for 4 h. Further on, the membranes were washed with distilled water until neutral pH was reached. After purification, the cellulose membranes were dried at 55 °C in an oven to constant weight. The productivity of BNC was quantified, based on the dry weight of the BNC pellicles, collected at the end of the 7-day cultivation and expressed in grams per liter. Experiments were performed in quadruplicate and the results are given as means.

### Scanning electron microscopy

Morphology and microstructural features of the membranes were investigated, using the scanning electron microscope (SEM) FEI QUANTA 250 FEG, operating at 2 kV, magnification ×5000, ×20,000, and ×40,000. Prior to the analysis, BNC membranes were purified according to the methodology described by Fang et al. (Fang et al. 2015). The pellicles were freeze-dried in Christ Alpha model 1-4 LSC plus. Before the SEM analysis, the samples were coated with gold/palladium. After the observation of three biological replicates, representative micrographs were taken in triplicates for each magnification. The diameters of fibers and pores were determined with the *Makroaufmassprogram*, with 50 fibers and pores from the SEM micrographs.

### Sample preparation and fluorescence microscopy

The cells were grown at 30 °C for 48 h in SH medium with the appropriate antibiotics. A small portion of a culture was dropped on the slide glass and cover glass was placed. Cells were observed under a Nikon Eclipse TS100 microscope (Nikon Instruments, Tokyo, Japan). The microscope settings were as follows: excitation at 340–380 nm, emission at 435–485 nm.

### RNA isolation and quantitative reverse transcription PCR analysis

*K. hansenii* ATCC 23769 wild-type strain, pTI99, *motA+*, and *motB+* were cultured in 10 mL test tubes containing 5 mL of a SH medium at 30 °C under static conditions for 4 days. One percent (*v*/*v*) cellulase (from *Trichoderma reesei* ATCC 26921, Sigma-Aldrich, Germany) was added to the culture and incubated for 2 h in 30 °C. Total RNA was extracted and purified using the GeneMatrix Universal RNA Purification Kit (EURx, Poland). RNA samples were reverse transcribed with Fast SG qPCR Master Mix (2x) kit (EURx, Poland). Primers (Table S2) were designed using the NCBI’s Primer-BLAST software (National Center for Biotechnology Information). RT-qPCR was carried out using a CFX96TM detection system (Hercules, Bio-Rad, USA). The reverse transcription reaction was performed at 50 °C for 2 min and followed by conventional qPCR amplification cycles (95 °C for 10 min, 1 cycle; 94 °C for 15 s, 59 °C for 30 s, 72 °C for 30 s, 40 cycles), conducted in triplicate. Transcript quantities of each target gene were normalized to the reference *16S rRNA level*. The target gene expression levels were calculated using the Livak and Schmittgen equations (Livak and Schmittgen 2001):

1 $$ \Delta  \mathrm{CT}\ \left(\mathrm{sample}\right)=\mathrm{CT}\ \left(\mathrm{target}\ \mathrm{gene}\ \mathrm{for}\ \mathrm{sample}\right)\hbox{--} \mathrm{CT}\ \left(16\mathrm{S}\ \mathrm{rRNA}\ \mathrm{for}\ \mathrm{sample}\right) $$

2 $$ \Delta  \mathrm{CT}\ \left(\mathrm{wild}-\mathrm{type}\right)=\mathrm{CT}\ \left(\mathrm{target}\ \mathrm{gene}\ \mathrm{for}\ \mathrm{wild}-\mathrm{type}\right)\hbox{--} \mathrm{CT}\ \left(16\mathrm{S}\ \mathrm{rRNA}\ \mathrm{for}\ \mathrm{wild}-\mathrm{type}\right) $$

3 $$ \Delta  \Delta  \mathrm{CT}=\Delta  \mathrm{CT}\ \left(\mathrm{sample}\right)-\Delta  \mathrm{CT}\ \left(\mathrm{wild}-\mathrm{type}\right) $$

4 $$ \mathrm{Relative}\ \mathrm{gene}\ \mathrm{expression}={2}^{-\Delta  \Delta  \mathrm{CT}} $$

### Statistical analysis

All data were expressed as mean ± standard deviation (SD). The statistical analysis was calculated using Student’s *t* test, and statistical significance was set at *p < 0.05 and **p < 0.001 and extreme significance was set at ***p < 0.0001.

## Results

### Bioinformatical analysis of the target genes

**Fig. 1:**
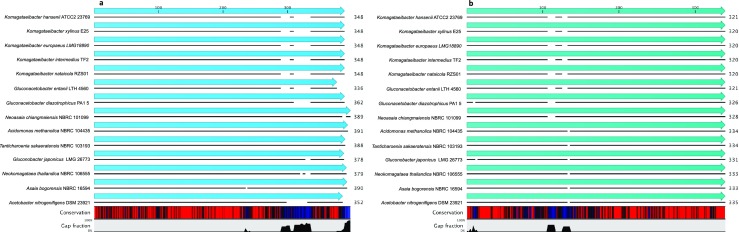
Multiple sequence alignment (MSA) of *motA* (**a**) and *motB* (**b**) genes. Figures generated using CLC Sequence Viewer

Based on genomic sequence of *K. hansenii* ATCC 23769 (Pfeffer et al. 2016), we have chosen two genes encoding proteins with homology to the MotA/ExbB/TolQ and MotB/ExbD/TolR proteins. The only sequences having homology with known motility-related genes were *motA* and *motB* homologs. *Komagataeibacter hansenii* ATCC 23769 *motA* and *motB* genes encode proteins of 348 and 321 amino acids, respectively. These two proteins are highly conserved among *Komagataeibacter* species, which suggests that they have an important cellular function. Moreover, the *motA* and *motB* genes were found to be located in the neighboring loci, possibly forming an operon in the *Komagataeibacter* species (Fig. S1). We observed that in different gram-negative species belonging to *Alphaproteobacteria* class, *motAB* genes are also located in their neighboring loci (Fig. 1). Such organization is consistent with the reports regarding the organization of *motA*-*motB* genes in the *Alphaproteobacteria* class (Liu and Ochman 2007a; Liu and Ochman 2007b). Interestingly, the N-terminal sequence of MotA protein and the C-terminal sequence of MotB protein are highly conserved among Gram-negative bacteria.

BlastP analysis of MotA revealed the high level of similarity to the amino acid sequences of MotA proteins,that *energize* flagellar rotation in bacteria with polar flagellum (Table 1).

**Table 1:** BLASTp search with the *K. hansenii* ATCC 23769 MotA and MotB sequences against the genomes from *Alphaproteobacteria* class

	Accession no.	Genome annotation	Organism	Coverage (%)	Identity (%)
MotA	WP_012553814	MotA/TolQ/ExbB proton channel family protein	*Gluconacetobacter diazotrophicus* PA1 5	99	68
	KXV29659	Flagellar motor protein MotA	*Gluconobacter japonicus* NBRC 103476	98	59
	WP_086640501	Flagellar motor protein MotA	*Acetobacter tropicalis* NBRC 101654	98	63
MotB	APO69134	OmpA/MotB domain protein	*Rhizobium gallicum* IE4872	98	46
	WP_012567007	Chemotaxis MotB protein	*Rhodospirillum centenum* SW	95	48
	WP_041039755	Flagellar motor rotation protein MotB	*Magnetospirillum magnetotacticum* MS-1	99	46

Knowing different roles of the paralogous proteins, we searched for sequence similarity and predicted transmembrane topology. Results of transmembrane domains prediction suggested 4 TM domains with large cytoplasmic domain between 2nd and 3rd TMs in MotA candidate (Fig. S2 a), which would make it more similar to MotA not ExbB/TolQ proteins (they possess 3 TM domains). Overall architecture of MotB (one TM domain, OmpA-like domain at C terminus, mostly periplasmic) again shows closer similarity to the stator protein then to the ExbD/TolR paralogs (Fig. S2 b).

Although, bacteria of the *Komagataeibacter* genus do not have flagellum, sequence-based predictions show that the investigated proteins probably form a proton pump. However, the protein partner of these proteins, to which energy is transformed, is to be elucidated experimentally and, moreover, it cannot be a FliH-like flagellum protein as it is absent in *Komagataeibacter*.

### *K. hansenii* ATCC 23769 *motA+* and *motB+* mutants’ phenotype—cell size and swarming motility

Based on the putative roles of MotA/ ExbB/TolQ and MotB/ExbD/TolR homologs (Hossain et al. 2005; Belas 2013; Teleha et al. 2013), we decided to limit *K. hansenii* ATCC 23769 mutant strains phenotype studies to cell lengths, motility, cellulose productivity, and pellicle microarchitecture.

**Fig. 2:**
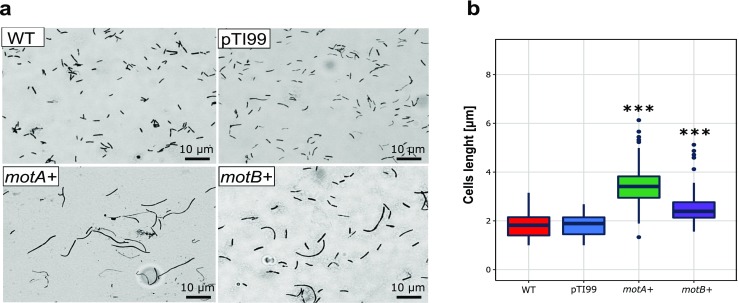
*Komagataeibacter hansenii* ATCC 23769 cell size estimation. **a** Example microphotographs of *K. hansenii* ATCC 23769 cells after crystal violet staining (magnification × 1000): wild-type, control with empty plasmid, and *motA+*, *motB+* cells are shown. **b** Mean cells lengths are given in micrometer, measured from 20 images taken every 24 h starting from 2nd to 7th day of incubation (120 fields of view for each sample). Error bars represent SD. Significance was determined using Student’s *t* test: (**) indicates *p* < 0.001 and (***) indicates *p* < 0.0001 compared to the wild-type strain

We created two overexpression mutants, *motA+* and *motB+* (Table 2). In order to gain a better understanding of the putative function of the tested genes, we analyzed phenotype of their overexpression mutants. Firstly, we checked the morphology and observed difference in cell behavior between the wild-type strain and its overexpression mutants (Fig. 2a). The cells of wild-type *K. hansenii* ATCC 23769 were 1.00–3.15 μm in length. Overall, cells *motA+* and *motB+* mutants were longer in comparison to cells of wild-type. The cells of *motA+* and *motB+* were 1.33–6.13 μm and 1.56–5.12 μm in length respectively (Fig. 2b). Most mutants’ cells occurred as elongated rods growing in long chains, with regions indicative of septum formation.

**Table 2:** Bacterial strains and plasmids used in this study

Strains/plasmids	Relevant characteristics	Reference/source
Strains		
*Eschericha coli* TOP10F’	*F’lac*I^q^*Tn10*(Tet^R^) *mcr*AΔ(*mrr-hsd*RMS-*mcr*BC) ϕ80*lac*ZΔM15 Δ*lac*X74 *deo*R *rec*A1 *ara*D139 Δ(*ara-leu*)7697 *gal*U *gal*K *rps*L (Str^R^) *end*A1 *nup*G	Invitrogen, USA
*Komagataeibacter hansenii*		
ATCC 23769	Wild-type strain	Hokkaido University, Japan
Plasmids		
pTI99	Creation pTI99-motA and pTI99-motB vectors	Hokkaido University, Japan
pTIE	Creation MotA-GFP, MotB-GFP vectors	Hokkaido University, Japan
pTI99-motA	*K. hansenii* motA+ overexpression mutant	This study
pTI99-motB	*K. hansenii* motB+ overexpression mutant	This study
pTIE-MotA-GFP	*K. hansenii* MotA-GFP overexpression mutant	This study
pTIE-MotB-GFP	*K. hansenii* MotB-GFP overexpression mutant	This study

We observed that overexpression of *motA* and *motB* genes caused an elongate cell phenotype under normal growth conditions. In some microorganisms, swarming motility is associated with cell elongation (Fraser and Hughes 1999). Furthermore, it has been shown that some bacteria become elongated during swarming (Little et al. 2018). In Gram-negative bacteria, the Tol-Pal complex is widely conserved and may have various physiological functions such as maintenance of cell envelope integrity, interaction with outer membrane porins, expression of lipopolysaccharide surface antigens and virulence factors, facilitation of infection by filamentous DNA phage, and improvement resistance to detergents (Cascales et al. 2000; Gaspar et al. 2000; Cascales et al. 2001). Filamentous phenotype implies a defect in cell division such that cells continue to grow in the absence of septation. Cells that seem to be elongated can also form by the failure of cell separation following successful division, consequently resulting in cells that are connected end-to-end in long chains (Kearns and Losick 2005; Patrick and Kearns 2008). Teleha et al. reported that *tolQ* and *tolR E. coli* overexpression mutants’ cells display an elongated phenotype, suggesting role of these genes in cell division (Teleha et al. 2013). Therefore, we decided to investigate the effect of *motA* and *motB* overexpression on *E. coli* TOP10F’ cell morphology. No differences were observed in cell morphology between the *motA+*, *motB+* mutants and the control with pTI99 and the wild-type strain (Fig. S3).

In order to test if cells with increased motility could form cellulose pellicles with loosened network, we performed motility assay for *K. hansenii* ATCC 23769 WT, control, and *motA+ motB+* mutants. Motility assay showed that *K. hansenii* ATCC 23769 is able to spread over moist surfaces in a process called “swarming.” Observation of the bacterial colonies spreading on the soft agar is commonly interpreted as presence of swarming motility in the tested microorganisms.

**Fig. 3:**
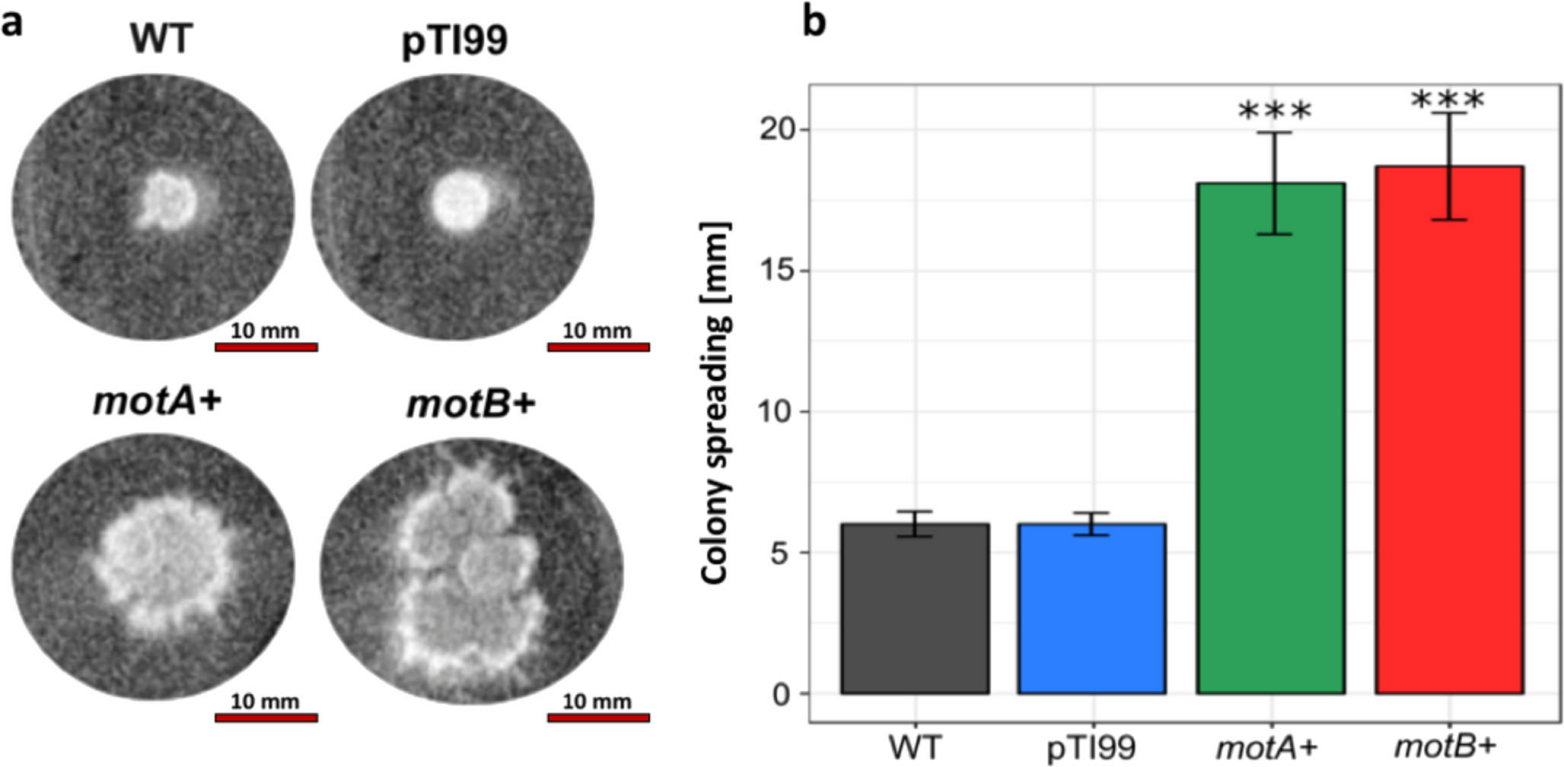
Motility assay results. **a** Example photograph of colonies formed by *K. hansenii* ATCC 23769 WT, control, and *motA+* and *motB+* on soft agar (0.3%). Images taken on 5rd day of incubation. **b** Changes in colonies’ size [mm] measured every 24 h, starting from 3nd and finishing on 5th day of incubation. Mean values were calculated from five biological replicates; error bars represent standard deviations. Significance was determined using Student’s *t* test: (***) indicates *p* < 0.0001 compared to the wild-type strain

The swarming motility of *motA+* and *motB+* mutants, control, and wild-type *K. hansenii* ATCC 23769 strain was analyzed over 3 subsequent days (Fig. 3a). The cells of the wild-type strain formed colonies with thin spreading edges, whereas the *motA+* and *motB+* cell mutants formed irregular spreading colonies on agar. Based on motility assay, we observed a threefold increase in speeding of mutant colonies in comparison to the WT and the controls (Fig. 3b).

Swarming typically involves collective movement of a dense population of bacteria cells. It is a common manner of motility that allows bacteria to rapidly colonize a surface, leading to the biofilm formation (Donlan 2002). In some microorganisms, swarming motility is associated with cell elongation (Fraser and Hughes 1999). Furthermore, it has been shown that some bacteria become elongated during swarming (Little et al. 2018).

### Characterization of BNC membranes produced by mutant strains

**Fig. 4:**
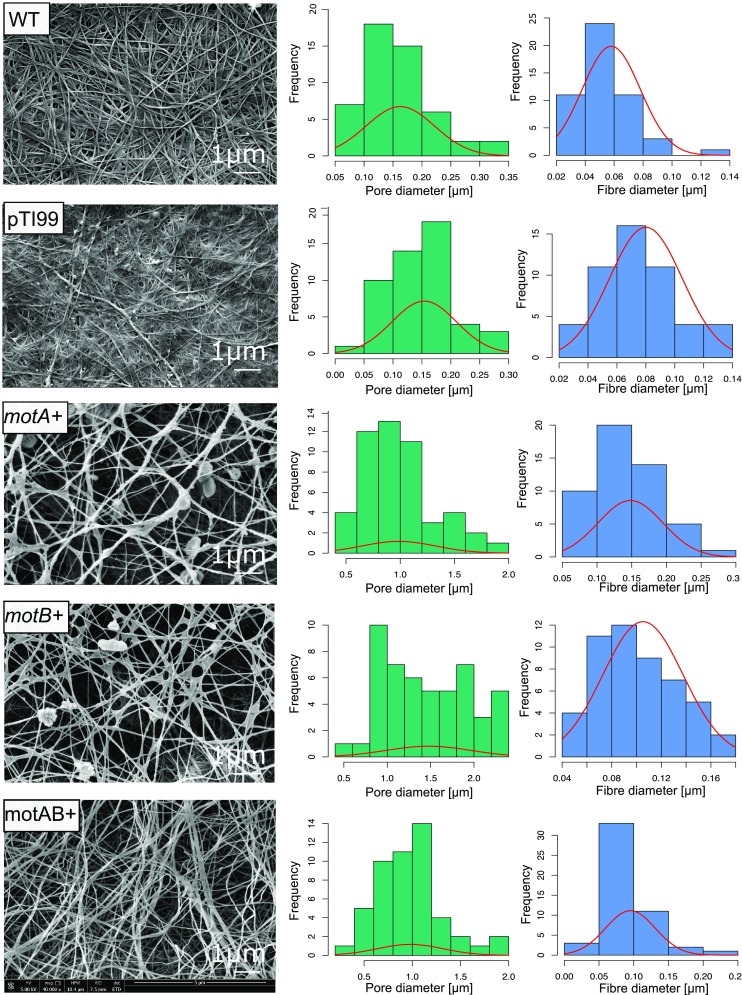
Characterization of BNC membranes: left column scanning electron micrographs (ETD detector, magnification × 40,000), middle column: pores diameters distribution (50 measurements for each sample), right column: fibers diameters distribution (50 measurements for each sample)

To understand the importance of the effect of overexpression of *motA* and *motB* genes on the structure of BNC membranes, we performed the SEM imaging. Both mutants produced membranes with significantly loosened network (Fig. 4, left column), which was confirmed by estimating the diameters of 50 pores (Fig. 4, middle column). Interestingly, the mutant *motB*+ produced membranes with pores almost 9 times larger when compared to the wild-type strain. The pore sizes of membranes produced by the wild-type *K. hansenii* ATCC 23769 and pTI99 control were 0.16 ± 0.06 and 0.15 ± 0.06 μm, and by *motA+* and *motB+* mutants were 0.99 ± 0.34 μm, and 1.48 ± 0.49 μm, respectively. We also investigated the relationship between motility, length of the bacterial cells, and the size of the fiber thickness in mutants and WT (Fig. 4, right column). *MotA+* and *motB+* mutants produced thicker fibers compared to the wild-type strain. An average cellulose fibers’ size in the WT strain and *motA+*, *motB+* mutants was about 0.06 ± 0.02, 0.148 ± 0.05, and 0.105 ± 0.03 μm, respectively.

High porosity is significant feature of 3-D biopolymer scaffolds used in tissue engineering because of its essential effects on cell growth. It is generally assumed that pore size of a scaffold may affect the cell adhesion and proliferation (Bäckdahl et al. 2006). Material obtained from mutants has a great application potential as a scaffold in tissue engineering due to the fact that it has significantly enlarged pores when compared to wild-type membranes and there are possibilities to control the pores sizes.

### Impact of bacterial motility and cell morphology on bacterial nanocellulose production

Before comparing the yields of bacterial nanocellulose, we examined the growth rates of *motA+* and *motB+* mutants over a period of 6 days to investigate the effect of *motA* and *motB* genes overexpression on the cell growth. We decided to monitor the growth rate of each culture by its colony-forming unit (CFU) since, under the static growth condition, cells producing cellulose are fixed in the cellulose membrane formed at the surface of the medium, which makes monitoring cell growth by optical density imprecise.

**Fig. 5:**
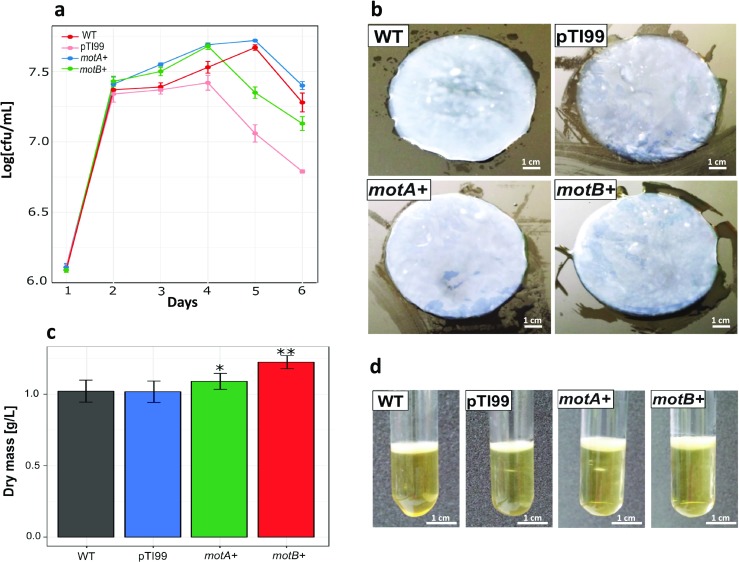
Characterization of *K. hansenii* ATCC 23769 WT, control with empty plasmid, and *motA+* and *motB+* strains. **a** Growth curve. **b** Morphology of a typical cellulose pellicle produced by *K. hansenii* ATCC 23769 WT and mutant strains after 7 days in 50 ml SH. **c** Bacterial nanocellulose productivity of *K. hansenii* ATCC 23769 WT and mutant strains, shown as pellicle dry mass after a 7-day incubation in 50 mL SH media. **d** Example photographs of culture in 5 mL SH medium after 7 days. Mean values were calculated from four biological replicates; error bars represent standard deviations. Significance was determined using Student’s *t* test: (*) indicates *p* < 0.05 and (**) indicates *p* < 0.001 compared to the wild-type strain

The growth curves of *Komagataeibacter hansenii* ATCC 23769 wild-type and its overexpression mutants showed that there were slight differences of growth of *motA+*, *motB+*, when compared with the wild-type strain. Up to day 4, the growth rates of *motA+* and *motB+* mutants were slightly higher than those of WT; however, at the end of day 6, all of the mutants and WT reached similar CFU values (Fig. 5a).

In order to investigate the efficiency in the production of BNC by mutants, wild type, pTI99, and *motA+*, *motB+* were grown for 3 days in 5 mL SH medium under static conditions; for overexpression mutants, ampicillin (200 μg/mL) was added, and then 5% of inoculum was transferred into 50 mL SH medium. After growth under static conditions for 7 days, BNC membranes were harvested and purified (Fig. 5b) and their dry weights were determined. The results shown in Fig. 5c, d revealed that the mutants produced more BNC, when compared with the wild-type. *MotA+* and *motB+* mutants produced 6% and 20% more cellulose respectively, in contrast to WT and pTI99. These results suggest that overexpression of *motA* and *motB* genes not only affected the morphology, but also the yield, of bacterial nanocellulose production.

There is no information explaining the impact of motility and cell length on BNC biosynthesis by *K. hansenii* ATCC 23769. Our results indicate that *motA, motB* genes may have an impact not only on the BNC structure but also on the cellulose yield. In the latest work, Basu et al. also noticed that there is a correlation between cell motility and cellulose yields (Basu et al. 2018). Several studies suggest that motility and cell size are involved in biofilm formation in many different microbes (O’Toole and Kolter 1998; Pratt and Kolter 1998; Choy et al. 2004; Merritt et al. 2007; Niba et al. 2007). It has been demonstrated that deletion mutant of *motA* was non-motile and displayed reduced ability to form biofilm (Hossain and Tsuyumu 2006). Further, it was reported that the *tonB*1 gene of *Pseudomonas aeruginosa* is not only required for transport of molecules involved in biofilm formation, but also in quorum sensing (Abbas et al. 2007).

### Subcellular localization of MotA and MotB proteins

**Fig. 6:**
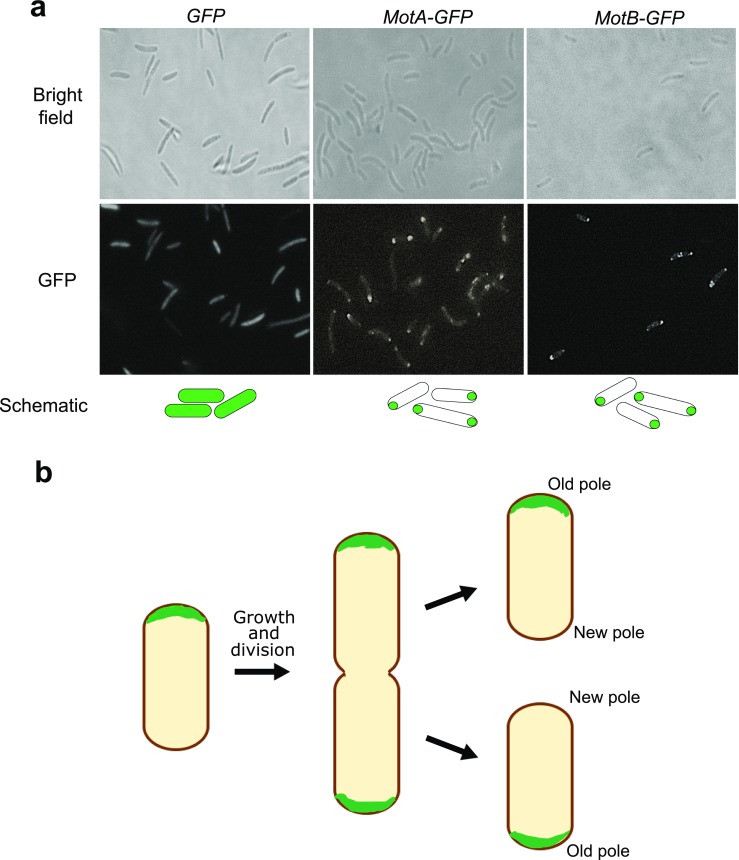
**a** Subcellular localisation of MotA and MotB proteins in *K. hansenii* ATCC 23769. **b** Possible bipolar to old-pole localization of MotA and MotB produced by a cell division event

To investigate the localization of MotA and MotB proteins in *K. hansenii* ATCC 23769 cells, we constructed expression systems for green fluorescent protein (GFP) fused MotA and MotB proteins. These C-terminal fused constructs were designated *MotA-GFP* and *MotB-GFP*, respectively. As expected, localization of *MotA-GFP* and *MotB-GFP* occurred at the cell pole (Fig. 6a). Moreover, we have observed that both MotA and MotB have a polar location when cells are short, whereas bipolar when cells are longer (Fig. 6b).

### Relative gene expression

**Fig. 7:**
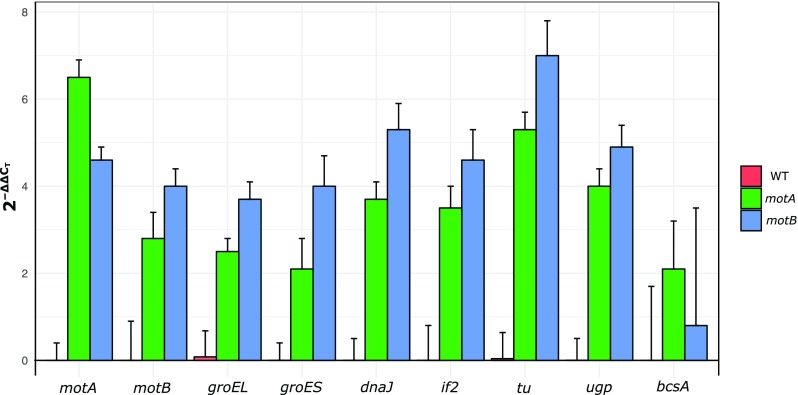
The relative expression of selected genes from qPCR data in *motA+* and *motB+* strains, where the expression levels in the *motA+* and *motB+* samples were normalized with the mean expression in wild-type samples. Samples were normalized to wild-type samples with the Livak method and *16S rRNA* gene was used as the reference gene. Mean values were calculated from three biological replicates; error bars represent standard deviations

In order to evaluate the influence of overexpression of the *motA* and *motB* genes in *K. hansenii* ATCC 23769 cells, RT-qPCR experiments were performed with the wild-type, *motA+* and *motB+* genes. In addition, transcript levels of molecular chaperons (*groES, groEL, dnaJ*), translation factors (*if2, tu*), and genes involved in the bacterial nanocellulose biosynthesis (*ugp, bcsA*) were evaluated (Fig. 7). The results of this analysis showed that the transcript levels of *motA* and *motB* were higher, both in *motA+* and in *motB+* mutants. Furthermore, the relative expression of *groES, groEL, dnaJ, if2*, and *tu* was increased in both mutants, nevertheless the highest in *motB+* mutant. In *motA+* and *motB+* mutants, the expression level of UDP-glucose pyrophosphorylase (*ugp*) was significantly upregulated, which suggests an enhanced UDP-glucose synthesis. Bacterial nanocellulose is synthesized from UDP-glucose by the action of cellulose synthase (*bcs*) multimeric enzyme (Jacek et al. 2019). *MotA+* and *motB+* are expressing *bcsA* at a 2.1 and 0.8-fold higher level respectively, in comparison to the wild-type strain.

## Discussion

Herein, for the first time, a new approach in modifying the structure of bacterial nanocellulose using genetic engineering tools is presented. We chose two genes encoding proteins which are homologs of MotA/ExbB/TolQ and MotB/ExbD/TolR proteins. Homologs of selected proteins are specific for Gram-negative bacteria and are involved in diverse activities. Two motility proteins MotA and MotB are essential for rotating the proton-driven flagellar motor (Kojima and Blair 2004). Furthermore, ExbB and ExbD participates in active transport mechanisms in TonB system, responsible mainly for macromolecules and complexes with ferric ions uptake but as well saccharides and DNA may be transported by these systems (Braun and Braun 2002; Maki-Yonekura et al. 2018). Third role of such systems was most frequently observed for TolA/TolQ homologs with its involvement in cell division regulation but by yet-unknown mechanism (Germon et al. 2001; Gerding et al. 2007).

Very high level of *motA* and *motB* sequence conservation between *Komagataeibacter* strains suggests involvement of the tested genes in yet-unknown but important mechanism, presumably connected with single cellulose chain secretion and further assembly in bacterial nanocellulose ribbon. Although bioinformatic studies have proven that the tested genes are more closely related to *motA* and *motB* homologs, we found them to be involved not only with bacterial movement but also in diverse activities, such as cell division, productivity of cellulose, and creation of 3-D cellulose network with larger pores and thicker fibers.

Our results suggested that cell size elongation influenced cellulose ribbons diameter and spatial arrangement as well. *MotA+* and *motB+* mutants produced membranes with loosened structure and thicker fibers compared to the wild-type strain. Therefore, this result gives a new line of evidence that cellulose fiber assembly occurring outside the *K. hansenii* ATCC 23769 cells is correlated with cell size. Cell elongation lead to fiber thickening. A possible explanation of this phenomenon could be in this manner. Bacteria grow by cell division. Cellulose networks are supposed to form together with cell division (Watanabett and Yamanaka 1995). It is considered that, in every cell division, a ribbon is divided into two in the horizontal direction; thus, this action is reiterated with every cell division and with every separation of cells, and these reiterations result in networks of cellulose ribbons (Yamanaka et al. 1989). The same effect with cell elongation induced by, e.g., chloramphenicol addition to the medium was previously described to induce loosening of cellulose network (Yamanaka et al. 2000).

Interestingly, *motA+* and *motB+* overexpression mutants showed significantly increased cellulose productivity as well. Several studies suggest that motility and cell size are involved in biofilm formation in many different microbes (O’Toole and Kolter 1998; Pratt and Kolter 1998; Choy et al. 2004; Merritt et al. 2007; Niba et al. 2007). It has been demonstrated that deletion mutant of *motA* was non-motile and displayed reduced ability to form biofilm (Hossain and Tsuyumu 2006). Despite the fact that both *motA* and *motB* play an essential role in the biofilm formation in Gram-negative bacteria, there is no evidence of the role of these genes in *Komagataeibacter* genus.

Localization of MotA-GFP and MotB-GFP occurred at the cell pole. Furthermore, we have observed that both MotA and MotB have a polar location when cells are short, while bipolar when cells are longer. Similar results were obtained in bacteria with polar flagella (Koerdt et al. 2009; Wang et al. 2013). In *Vibrio alginolyticus*, PomA and PomB proteins, which are MotA and MotB homologs, are localized to a cell pole dependent on the presence of the polar flagellum (Fukuoka et al. 2005; Kojima 2015). The polar localization of proteins is a crucial functional feature, since it is critical for correct performance of several essential cellular processes including growth and cell division as well as cell motility (Shapiro et al. 2002; Ryan and Shapiro 2003; Treuner-Lange and Søgaard-Andersen 2014). Similar localization of Mot proteins was observed for in bacteria with polar flagella (Koerdt et al. 2009). Since, bacteria from the *Komagataeibacter* genus do not have flagella, we suppose that this feature is not essential for BNC production and maybe was reduced during evolution.

The RT-qPCR results indicated that the overexpression of *motA* and *motB* genes in *K. hansenii* ATCC 23769 strain resulted in a higher expression level of *motA*, *motB*, *groEs*, *groEL*, *dnaJ*, *if2*, *tu*, *ugp*, and *bscA* genes. GroES/EL and DnaJ contributes to diverse cellular functions, including stress responses, motility, and pathogenesis (Shi et al. 1992; Susin et al. 2006). Translational factors are involved in many mechanisms, including replication, transcription, RNA processing, DNA repair, regulation of translation, malignant transformation, and regulation of cell growth and development (Caldas et al. 2000; Madison et al. 2012). Translation initiation factor (*if2*) and translation elongation factors (*tu*), in addition to their role in translation, might be implicated in protein folding and protection from stress. Several studies have reported upregulation of molecular chaperons and stress-responsive genes, e.g., by ethanol (Okamoto-Kainuma et al. 2004). Furthermore, the expression level of *ugp* and *bcsA* genes was upregulated in the *motA+* and the *motB+* mutants, which might explain the observed increase in the efficiency of BNC production. These observations are in agreement with the previous reports suggesting that MotA and MotB proteins are important in biofilm formation (Hossain and Tsuyumu 2006).

In this study, we show for the first time the functional role of MotA and MotB in the regulation of bacterial cellulose production and creation of porous BNC network. By control of *K. hansenii* motility and cell size, it is possible to direct cells to produce well-defined three-dimensional scaffold for tissue regeneration and repair. Therefore, we believe that it is necessary to discover and examine other genes related to cell division or motility, as this may in the future allow obtaining cellulose with desirable properties depending on the application.

## Electronic supplementary material

ESM 1 (PDF 3376 kb)
