# Supplementary material for: Structural changes of bacterial nanocellulose pellicles induced by genetic modification of Komagataeibacter hansenii ATCC 23769
Source: Appl Microbiol Biotechnol. 2019 Apr 29;103(13):5339–53. doi: 10.1007/s00253-019-09846-4 (PMC6570709; doi:10.1007/s00253-019-09846-4)
Supplement: Supplementary file 1 — (PDF 3376 kb) [file 253_2019_9846_MOESM1_ESM.pdf]

Applied Microbiology and Biotechnology

Structural changes of bacterial nanocellulose pellicles induced by genetic modification of *Komagataeibacter hansenii* ATCC 23769

Paulina Jacek[*], *Małgorzata Ryngajłło, Stanisław Bielecki*[1] *

Institute of Technical Biochemistry, Lodz University of Technology, B. Stefanowskiego 4/10, 90-924 Lodz, Poland

E-mail addresses: paulina.jacek@edu.p.lodz.pl, stanislaw.bielecki@p.lodz.pl

* Corresponding authors

Supplementary materials

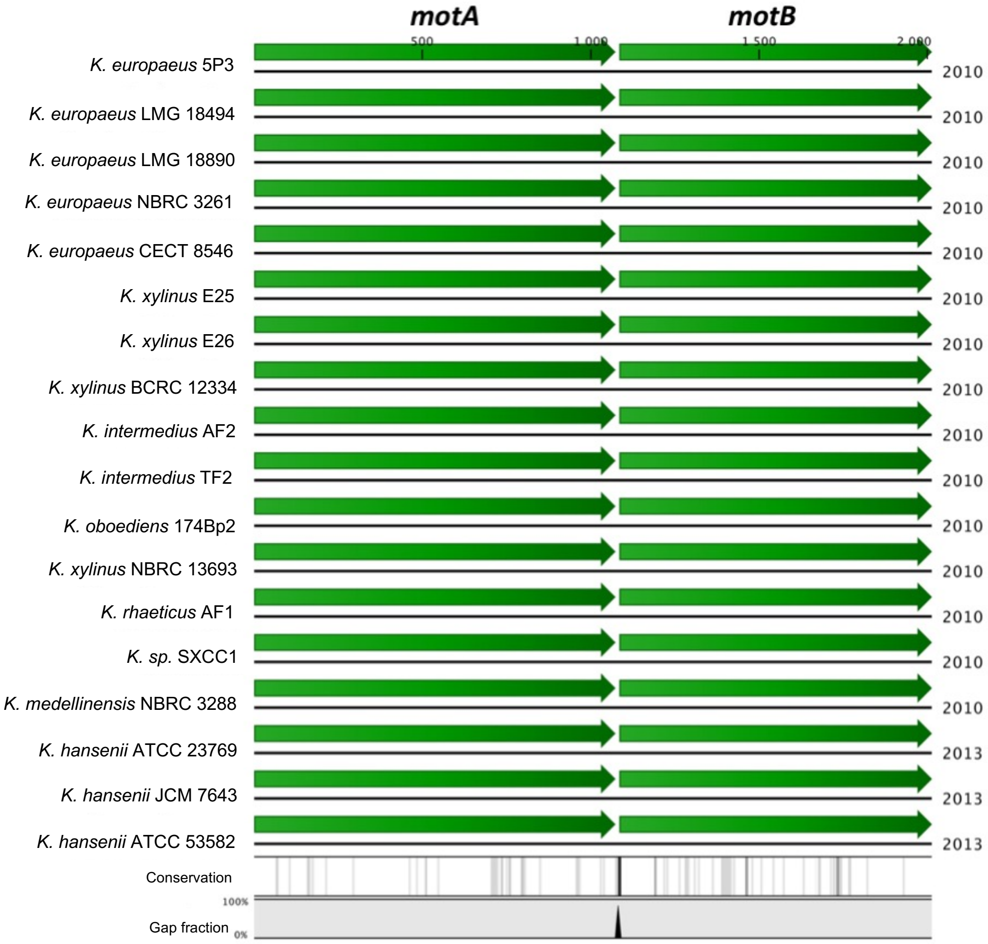

Fig. S1 Multiple Sequence Alignment (MSA) of *motA* and *motB* nucleotide sequence among the *Komagataeibacter* species. Figures generated using CLC Sequence Viewer.

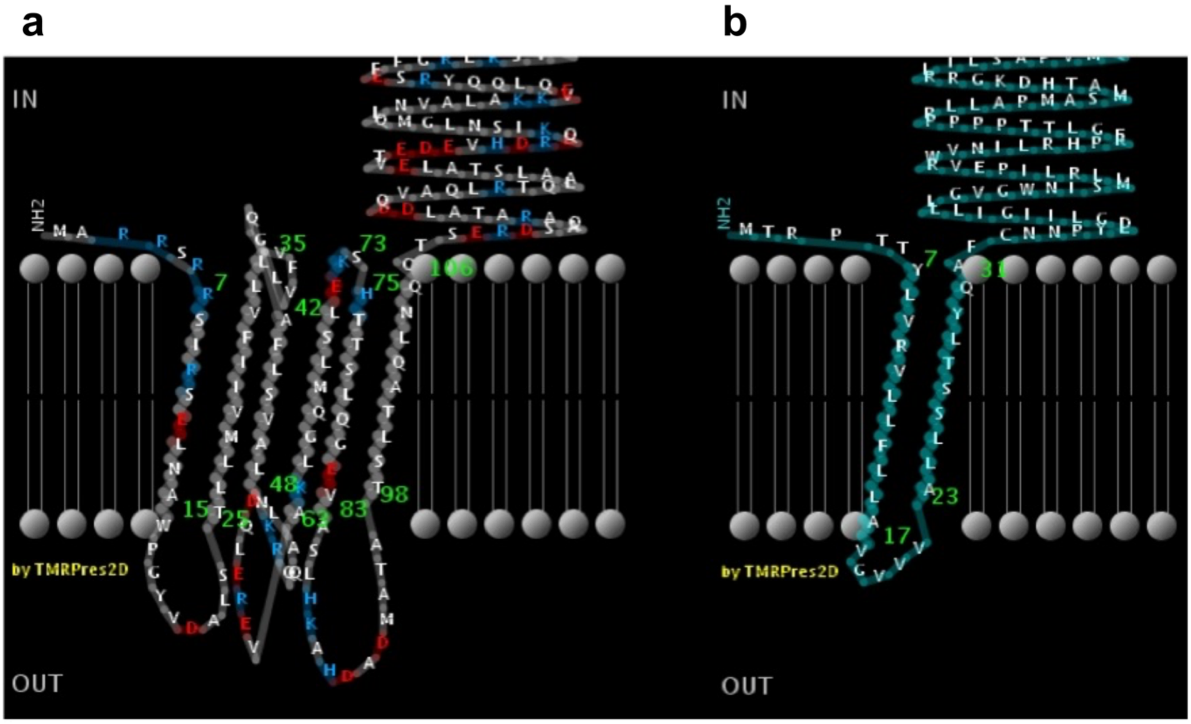

Fig. S2 Transmembrane topology predictions performed with TMHMM version 2.0 for *K. hansenii* ATCC 23769 MotA (a) and MotB (b). The periplasmic space is in the upper and the external surface is on the bottom of the drawings. Visuals were generated using TMRPres2D (Spyropoulos et al. 2004).

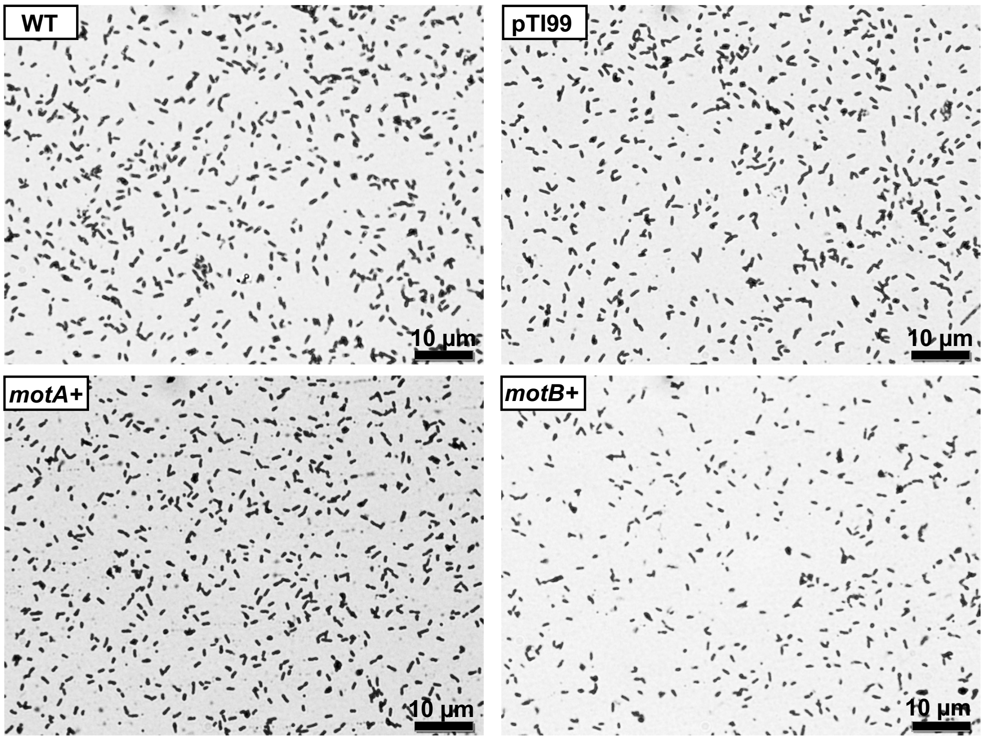

Fig. S3 Example microphotographs of *E. coli* TOP10F' cells after crystal violet staining (magnification 1000×): wild-type, control with empty plasmid and overexpression mutants *motA*+, *motB*+ cells are shown.

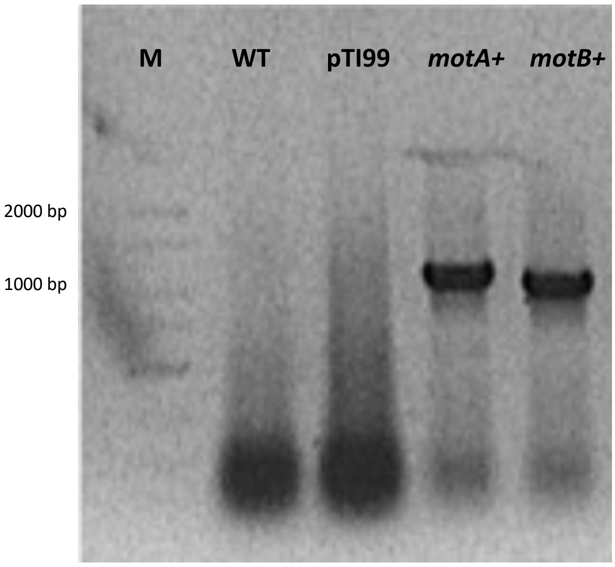

Fig. S4 Results after agarose gel electrophoresis of colony PCR products of after overexpression *K. hansenii* ATCC 23769 WT (line 2), control with pTI99 (line 3), *motA*+ (line 4) and *motB*+ (line 5).

Table S1 Primers used in this study.

Primer	Sequence (5’-3’)
pTI-motA_for	CGCGGATCCGTGACACGACCGACGACCTA
pTI-motA_rev	CCCAAGCTTTCAGGAAGCGGAGTGGTTCG
pTI_motB_for	CGCGGATCCATGGCGCGCCGTTCCC
pTI_motB_rev	CCCAAGCTTTCAGCGATCCGTCAGGCGGA
pTI_F	AAAATCTTCTCTCATCCG
pTI_R	ACACAGGAAACAGACCAT
motA_gfp_F	CCGGAATTCGTGACACGACCGACGACCTAT
motA_gfp_R	CGCGGATCCGGAAGCGGAGTGGTTCGTTC
motB_gfp_F	CGAGCTCATGGCGCGCCGTTCCCGTCG
motB_gfp_R	CGCGGATCCGCGATCCGTCAGGCGGAATTCGA

Table S2 Sequences of primers used in reverse transcriptase quantitative PCR analysis.

Primer	Sequence (5’-3’)
16srRNA_fw	TTGACCTTAAGCCGGTGAGC
16SrRNA_rv	TTACGACTTCACCCCAGTCG
F_motA_RT	TACGCTGTACCAGGCGTTTT
R_motA_RT	CGCAGGACCATGGAGACATT
F_motB_RT	CGCGATAACGGTGGTGAAGA
R_motB_RT	GTGCCATTATCCAGCGGTTG
F_groEL_RT	ATGGCTGCCAAGGACGTAAA
R_groEL_RT	CCTTCGGACCCAGTGTTACC
F_groES_RT	GCGAGGTTATCTCGGTCGG
R_groES_RT	ACCATTTGCCGAACAGCAC
F_dnaJ_RT	GGCGATACCGAGTCCGAAAA
R_dnaJ_RT	ATGGCCAAAGCGGTCGTAG
F_IF2_RT	GATCACGGCAAGACCTCGTT
R_IF2_RT	GTCACCTGATACGCACCGAT
F_tu_RT	CGAAATCGTTGGTCTGCGTC
R_tu_RT	ACCGATGTTGTCACCAGCTT
F_ugp_RT	TGACGTTGTGCAGAGCAAGA

R_ugp_RT	TTTCACGCGGAACCTCAGTC
F_bcsA	TACCTGCTGAACTCCGCTTG
R_bcsA	TGCGATGACTGTTGCGTTTC
